# No detectable fitness cost of infection by cell-fusing agent virus in *Aedes aegypti* mosquitoes

**DOI:** 10.1098/rsos.231373

**Published:** 2024-01-10

**Authors:** Yasutsugu Suzuki, Takahiro Suzuki, Fuminari Miura, Jerica Isabel L. Reyes, Irish Coleen A. Asin, Wataru Mitsunari, Mohammad Mosleh Uddin, Yu Sekii, Kozo Watanabe

**Affiliations:** ^1^ Center for Marine Environmental Studies (CMES), Ehime University, Bunkyo-cho 3, Matsuyama, Ehime, Japan; ^2^ Graduate School of Science and Engineering, Ehime University, Bunkyo-cho 3, Matsuyama, Ehime, Japan; ^3^ Faculty of Engineering, Ehime University, Bunkyo-cho 3, Matsuyama, Ehime, Japan; ^4^ Centre for Infectious Disease Control, National Institute for Public Health and the Environment (RIVM), Bilthoven, the Netherlands; ^5^ Department of Biochemistry and Molecular Biology (BMB), Faculty of Life Science, Mawlana Bhashani Science and Technology University (MBSTU), Santosh, Tangail, Bangladesh

**Keywords:** mosquito, insect-specific virus, cell-fusing agent virus, fitness cost, behaviour, tolerance

## Abstract

*Aedes* mosquitoes are well-known vectors of arthropod-borne viruses (arboviruses). Mosquitoes are more frequently infected with insect-specific viruses (ISVs) that cannot infect vertebrates. Some ISVs interfere with arbovirus replication in mosquito vectors, which has gained attention for potential use against arbovirus transmission. Cell-fusing agent virus (CFAV), a widespread ISV, can reduce arbovirus dissemination in *Ae. aegypti*. However, vectorial capacity is largely governed by other parameters than pathogen load, including mosquito survival and biting behaviour. Understanding how ISVs impact these mosquito fitness-related traits is critical to assess the potential risk of using ISVs as biological agents. Here, we examined the effects of CFAV infection on *Ae. aegypti* mosquito fitness. We found no significant reduction in mosquito survival, blood-feeding behaviour and reproduction, suggesting that *Ae. aegypti* is tolerant to CFAV. The only detectable effect was a slight increase in human attraction of CFAV-infected females in one out of eight trials. Viral tolerance is beneficial for introducing CFAV into natural mosquito populations, whereas the potential increase in biting activity must be further investigated. Our results provide the first insight into the link between ISVs and *Aedes* mosquito fitness and highlight the importance of considering all aspects of vectorial capacity for arbovirus control using ISVs.

## 1. Introduction

*Aedes* mosquito species, primarily *Aedes* (*Ae*.) *aegypti*, transmit several medically important arthropod-borne viruses (arboviruses) such as dengue virus (DENV), Zika virus (ZIKV) and chikungunya virus (CHIKV). In addition to these human pathogens, mosquitoes are infected with insect-specific viruses (ISVs), which are only infectious to insects but not to vertebrates. A recent study that reviewed 175 mosquito virome studies between October 2000 and February 2022 suggested that ISVs are more abundant than arboviruses in mosquito populations [1]. Although the existence of hundreds of new ISVs has been suggested by virome analysis in mosquitoes, few studies characterized the biological features of the identified ISVs. The only characteristic that is relatively well-tested on ISVs is the effect on arbovirus replication in mosquito vectors [2–14]. The presence of particular types of ISVs in mosquitoes has been shown to reduce arbovirus replication *in vitro* [7,8,12–14] and/or *in vivo* [6,7,11]. These studies have suggested that ISVs could be used as biological agents against arbovirus transmission from mosquitoes to humans.

Cell-fusing agent virus (CFAV; *Flaviviridae*, *Flavivirus*) is one of the few ISVs showing an anti-arbovirus effect in *Ae. aegypti in vivo*. It has been shown to inhibit DENV-1 and ZIKV dissemination in *Ae. aegypti* mosquitoes intrathoracically injected with CFAV [3]. CFAV was the first isolated ISV from *Ae. aegypti* cell lines in 1975 [15]. Since its discovery and sequencing, CFAV has been detected or isolated from the field-collected *Ae. aegypti* mosquitoes across different continents including Africa, Asia, Australia, North and South America [16–19]. Laboratory colonies of *Ae. aegypti* can also maintain CFAV infections [3,20]. A recent study has shown that CFAV can be venereally transmitted from infected males to uninfected females in adult stages but not vice versa [21]. The same study observed highly efficient maternal and paternal transmission. These observations suggest that CFAV spreads its infections mainly by vertical transmission in nature. Horizontal transmission during larval stages has yet to be explored. Virome studies suggested that CFAV is one of the most abundant ISVs in *Aedes* mosquitoes [1]. The availability of the CFAV isolates and naturally infected mosquito colonies allowed for characterizing their biological features such as evolutionary history or how ISVs maintain their infections in mosquito populations [16,21,22].

Arbovirus transmission occurs through the bites of mosquitoes that are systemically infected with the virus. After the intake of the infectious blood meal, the virus traverses through mosquito organs in several days to become transmissible, a phase called extrinsic incubation period (EIP) [23,24]. The viral load and EIP in the mosquito vector are critical factors in determining the efficacy of arbovirus transmission. In addition, mosquito population size, lifespan, and blood-feeding behaviour also largely influence the vectorial capacity [25]. The potential use of ISVs to control arbovirus transmission requires further understanding of how ISV infections impact mosquito fitness and alteration of behaviour. However, little is known about the potential effects of ISV infections on mosquito fitness including survival and blood-feeding behaviour. Unlike arboviruses, ISVs are known to infect not only female mosquitoes but also males in nature because they are considered to be primarily maintained by vertical transmission. The fitness costs in male mosquitoes may contribute to the population size of mosquito vectors in the long term. In addition, investigating viral infections in male mosquitoes could provide great insight into mosquito-virus interactions to better understand how antiviral immunity has been established or to identify the host factors for virus infections.

In this study, we investigated the effects of CFAV infection on the fitness of *Ae. aegypti* mosquitoes particularly, its survival rate, blood-feeding behaviour, fecundity, and fertility because these factors heavily affect the arbovirus transmission potential of the mosquitoes [23]. To assess these fitness costs, we intrathoracically inoculated a CFAV isolate into CFAV-free *Ae. aegypti* adults. We observed that CFAV infection did not have significant impacts on the mosquito survival rate, blood-feeding behaviour and reproduction. Our overall results provide the first experimental evidence that *Ae. aegypti* is highly tolerant to CFAV infection, which provides valuable insight towards using ISVs for arbovirus control strategies.

## 2. Material and methods

### 2.1. Cell culture and virus production

C6/36 cell line (ATCC CRL-1660) derived from *Ae. albopictus*, which was confirmed as CFAV-free (electronic supplementary material, figure S1), was maintained at 28°C in L-15 Leibovitz's medium (Thermo Fisher Scientific, USA) supplemented with 10% fetal bovine serum (FBS; HyClone), 1% nonessential amino acids (Gibco), 2% tryptose phosphate broth (Sigma, USA), and 1% penicillin-streptomycin (Thermo Fisher Scientific, USA).

A wild type CFAV isolate (LR596014, European Nucleotide Archive) was kindly provided by Louis Lambrechts, Institut Pasteur, France. The CFAV stock was amplified in C6/36 cells and the supernatant was used for mosquito infection *in vivo*. To quantify CFAV RNA copies, the non-structural gene 3 (NS3) was amplified with primers containing the T7 promoter sequence followed by reverse transcription with MEGAscript T7 Transcription Kit (Thermo Fisher Scientific, USA) to create a standard curve for CFAV. The copy number of CFAV RNA was determined by reverse transcription-quantitative polymerase chain reaction (RT-qPCR) based on a serial dilution of the CFAV NS3 RNA with PrimeScript RT reagent Kit (Perfect Real Time, Takara, Japan) using random hexamers and iTaq Universal SYBR Green Supermix (Bio Rad, USA). The primer sequences were listed in electronic supplementary material, table S1. To accurately calculate CFAV RNA copies in the virus stock, the CFAV stock was treated with 10 units of RNase ONE Ribonuclease (Promega, USA) for 30 min at 37°C to remove naked RNA before RNA extraction.

### 2.2. Mosquitoes

We used a laboratory colony of an *Ae. aegypti* strain that was previously engineered to remove a CFAV-derived endogenous viral element (EVE) and avoid any antiviral effect of this EVE against CFAV [23]. The CFAV EVE-less strain was derived from an isofemale line of *Ae. aegypti* originating in Kamphaeng Phet Province, Thailand [24]. The mosquitoes were reared in an environmental chamber conditioned at 28°C, 80% relative humidity and a 12 h light: 12 h dark cycle. Adult mosquitoes were maintained with permanent access to a 10% sucrose solution.

### 2.3. CFAV infection in *Ae. aegypti* adults

At four to seven days after emergence, *Ae. aegypti* adult females and males were intrathoracically injected with 3.24 × 10^4^ genome copies of CFAV (50 nl of CFAV stock) with a nanoinjector (Nanoject III, Drummond Scientific, Broomall, PA, USA). For mock infection, 50 nl of the supernatant of C6/36 cells was used. Following the injection, mosquitoes were incubated at 28°C, 80% relative humidity and a 12 h light: 12 h dark cycle with constant access to a 10% sucrose solution and subjected to each experiment described below.

### 2.4. CFAV growth curve in the infected *Ae. aegypti* adults

*Ae. aegypti* female and male adults injected with CFAV were collected at 0, 2, 5, 7, 10, 15, 20, 25, 30 and 35 day post injection (DPI). Due to the lifespan, the male mosquitoes were sampled until 30 DPI. Total RNA was extracted from individual mosquitoes using NucleoSpin RNA (Macherey-Nagel, Germany). To quantify CFAV RNA copies, RT-qPCR was performed with PrimeScript RT reagent Kit (Perfect Real Time) and iTaq Universal SYBR Green Supermix as described above.

### 2.5. Mosquito survival curves

To account for mosquito mortality due to damage of intrathoracic injection, the number of dead mosquitoes on day 1 was excluded from generating the survival curve (at least 30 surviving mosquitoes per treatment were subjected to the analysis). CFAV- or mock-injected mosquito survival was recorded daily. Two independent experiments were performed. For each experiment, we conducted a non-parametric survival analysis and obtained Kaplan-Meier estimators of survival rates for both groups (i.e. control and CFAV-injected).

### 2.6. Human arm proximity assay

Mosquito attraction rate to the human arm was examined by the assay adapted from the one previously described [25]. Briefly, female mosquitoes were stored in a 17.5 × 17.5 × 17.5 cm cage (BugDorm-4E1515) 7 days after CFAV or mock injection. Six human volunteers (females and males, age range: 20–50) joined this assay. Volunteers' arms were placed underneath the cages containing CFAV- or mock-injected mosquitoes (30 adult females) with 2 cm-distance. The assay was performed three times for each volunteer. The mosquito behaviour was recorded by a video camera from above the cage for 5 min. 10 images were captured every 10 s, from 3 min 30 s to 5 min of the video. We counted the number of mosquitoes that approached the volunteers' arms and landed on the bottom of the cage. This experiment was independently conducted three times with two volunteers per experiment.

### 2.7. Artificial blood-feeding, fecundity and fertility

CFAV- or mock-infected mosquitoes were allowed to access 37^o^C-warmed rabbit blood for 15 min using an artificial blood-feeding system (Hemotek, UK). The blood-fed mosquitoes were counted and placed individually into fly vials containing cotton with water-wet paper on top for oviposition and with access to a 10% sucrose solution. At day 7 after blood-feeding, the eggs laid on the paper were counted and transferred to a 12-well plate containing 2 ml water. The number of hatched larvae was counted 5 days after the egg transfer.

### 2.8. Statistical analysis

The mosquito survival curve was analysed by the Kaplan-Meier method. The difference in survival curves between two groups was tested by the Log-rank test, where the null hypothesis is that there is no difference between those two. The Mann-Whitney U test was applied to compare the difference between CFAV- and mock-infected groups in the proportion of attracted mosquitoes to human arms, the number of eggs laid and the hatch rate. Comparison of the number of blood-fed mosquitoes between CFAV- and mock-infected individuals was conducted by Chi-square test. These analyses were performed using GraphPad 9 Prism software and {survival} package in R [26].

## 3. Results and discussion

### 3.1. CFAV established persistent infection in *Ae. aegypti* females and males

**Figure 1.**
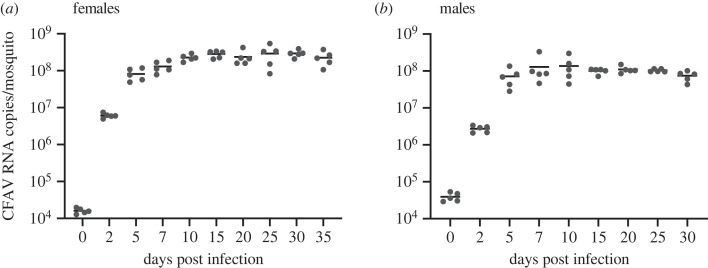
CFAV replicates in *Ae. aegypti* female and male adults. CFAV RNA levels were monitored in *Ae. aegypti* females (*a*) and males (*b*) intrathoracically injected with CFAV at the different time points indicated. Each dot represents an individual mosquito. Bars show mean.

To monitor the long-term CFAV replication kinetics in both female and male mosquitoes, *Ae. aegypti* adults were intrathoracically injected with CFAV. In both females and males, we observed that CFAV RNA copies were drastically increased within 2 DPI and reached a plateau after 7–10 DPI (figure 1). The highest levels of CFAV RNA lasted until 30 or 35 DPI, which is the latest time point examined, in males or females, respectively. This observation suggested that CFAV established persistent infections in *Ae. aegypti* adults for almost their entire lifespan. We also observed similar replication kinetics of CFAV in *Ae. aegypti* adult females and males. This observation implies that *Ae. aegypti* mosquitoes mount equivalent expression levels of host factors such as receptors necessitated for CFAV infections, regardless of sex. Most studies on viral kinetics in mosquitoes have focused on arbovirus infections in adult females and in a relatively short term such as 7 or 14 DPI. A few studies assessed the long-term viral kinetics of both ISVs and arboviruses in *Aedes* mosquito females for 21 DPI with Palm Creek virus (PCV, an ISV), and 20 or 32 DPI with DENV [2,27,28]. These studies showed that viral RNA levels remained high until the end of their experiment, which is consistent with what we observed using CFAV and *Ae. aegypti* adults. All of these suggest that *Aedes* mosquitoes would have a particular mechanism to establish persistent infections to broad types of viruses, and that makes the mosquitoes an efficient arbovirus vector.

### 3.2. No significant impact of CFAV infection on the lifespan of *Ae. aegypti* mosquitoes was found

**Figure 2.**
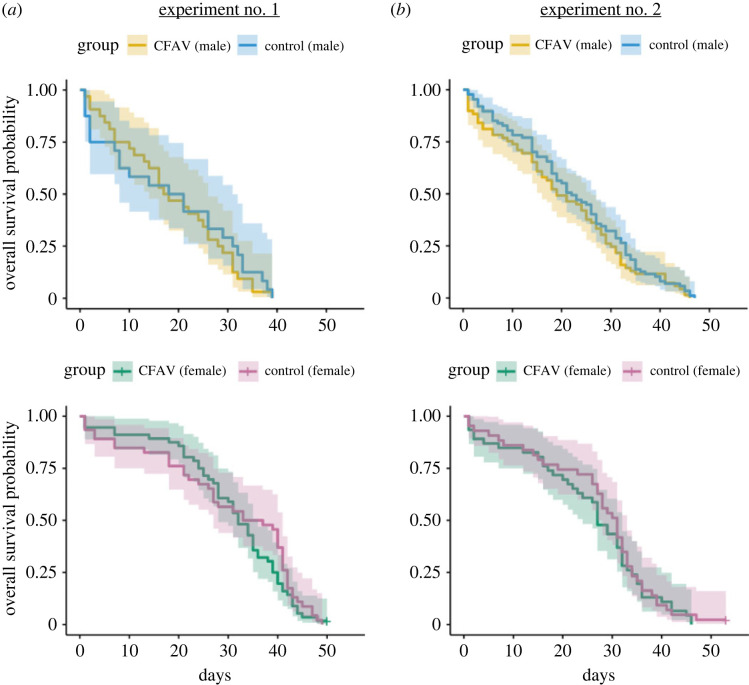
Kaplan–Meier curves for CFAV- or mock-infected *Ae. aegypti* mosquito survival. Two independent experiments were performed; Experiment no. 1 (*a*) and no. 2 (*b*). Yellow and blue indicates CFAV and mock infection, respectively in males, upper panels. Green and purple indicates CFAV and mock infection, respectively in females, lower panels. Survival estimates with 95% confidence intervals are shown as the shaded area of each corresponding colour.

To assess the potential impact of CFAV infection on the mosquito survival rate, we examined the mortality rate of CFAV- or mock-injected *Ae. aegypti* females or males. CFAV infection did not significantly alter the mosquito survival rate in both females and males during their lifespan in two independent experiments (figure 2) (Log-rank test: *p*-value = 0.4 for females and 0.08 for males). This negligible effect on adult survival is less likely because of the clearance of CFAV since we detected high levels of CFAV RNA until 30 or 35 DPI (figure 1), suggesting that both female and male mosquitoes can cope with CFAV infection with minimum effects on their lifespan.

Our result is consistent with previous studies that *Aedes* adult females are highly tolerant to viral infections. Although few studies examined the impact of ISV infections on mosquito survival, there are several reports using the naturally infected *Aedes* or *Culex* mosquito colonies with ISVs including *Aedes* flavivirus (AEFV), CFAV or *Culex* flavivirus (CxFV) which have implied that these ISV infections do not induce high mortality [11,20,21,29,30]. A recent study reported that *Cx. pipiens* adult males injected with an ISV, Culex Y virus (CYV), showed a mild but statistically significant decrease in their lifespan which was not observed in female mosquitoes [31]. One exceptional type of ISV that induces lethal effects in mosquitoes, particularly in larval stages, is densovirus isolated from *Aedes* mosquito species [32–34]. For instance, *Ae. albopictus* densovirus 2 (AalDV2)-exposed larvae of both *Ae. aegypti* and *Ae. albopictus* showed high mortality rate with unknown mechanisms [34]. On the other hand, the surviving adults were also highly infected with AalDV2, implying that severe pathogenicity would not be induced in the adult stages. Arboviruses including DENV, ZIKV, or CHIKV have been generally shown to slightly reduce the longevity of *Aedes* adult female mosquitoes although the fact remains that *Aedes* mosquitoes transmit those viruses [35–41]. Higher mortality is found from 7–10 DPI in most cases, which is later than the typical EIP of arboviruses [42–45]. The magnitude of effect varies considerably depending on the combination of mosquito-virus species and strains; even so, no detectable mortality has been reported [37].

The mechanism behind this tolerance remains to be elucidated. A few studies suggested that mosquito immune system such as RNA interference (RNAi) pathway contributes to establish this tolerance against arbovirus infections [42,46,47]. RNAi pathway could explain our observation of this tolerance of *Ae. aegypti* against CFAV infection. Although the impact of ISVs on the mosquito survival has not been well studied, ISVs including CFAV would be a great tool to dissect the relationship between host immunity and the viral tolerance.

### 3.3. Human host-seeking behaviour was rarely affected by CFAV infection

**Figure 3.**
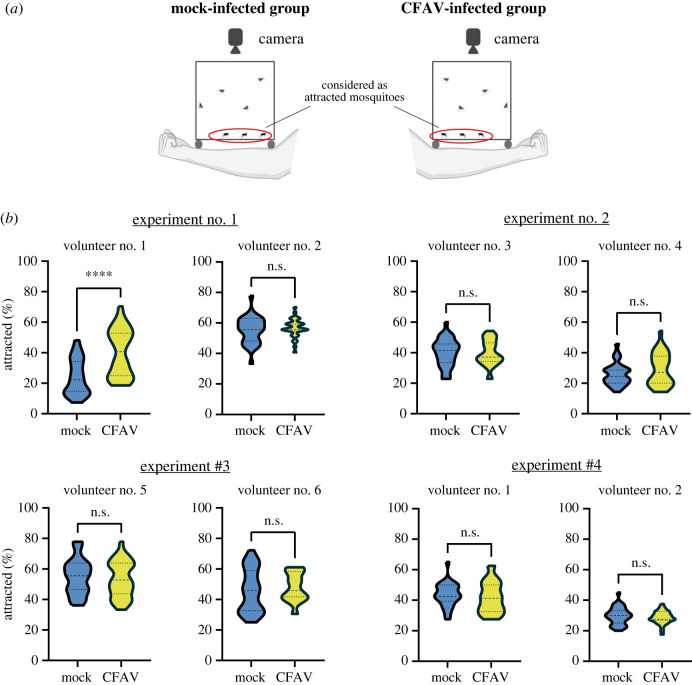
Human-host seeking assay. (*a*) Schematic representation of human arm proximity assay. Volunteers’ arms were placed underneath the cage containing CFAV- or mock-infected mosquito females (*n* = 40 per cage). The mosquito behaviour was recorded as a movie by a camera. The mosquitoes that landed next to the arms were considered attracted. (*b*) The proportion of attracted mosquitoes (%) to a human arm. Four experiments were performed on different days and with mosquito batches. Each experiment had two human volunteers. The assay with a volunteer was repeated three times. Asterisks show statistical significance (Mann-Whitney *U* test; ****p < 0.0001; ns, not significant).

To evaluate whether CFAV infection affects the human host-seeking ability of *Ae. aegypti*, we used an assay human arm proximity assay. In this assay, the mosquitoes that landed next to human arms were considered attracted individuals (figure 3*a*). Through all the experiments, the attraction rates varied by approximately 7–80% between experiments (figure 3*b*). Overall, we did not observe significant effects of CFAV infection on the human host attraction, except for Volunteer #1 in Experiment #1. In this experiment, we found that CFAV-infected mosquitoes were attracted more to the host compared to the mock-infected group (figure 3*b*; Experiment #1, Volunteer #1). However, this increase in attraction in the CFAV-infected group was not detected in another experiment with the same volunteer (figure 3*b*; Experiment #4, Volunteer #1). The range of attraction rate of Volunteer #2 also differed from Experiment #1 (figure 3*b*; Experiment #1 and #4, Volunteer #2). These observations suggest that the increase of attraction depends on the physiological condition of the human host and/or *Ae. aegypti* mosquitoes. It is well known that mosquitoes use carbon dioxide (CO_2_), skin odorants, and temperature from the target hosts to detect blood sources (reviewed in [48]). CFAV infection might enhance the sensitivity to these cues, especially volatiles such as carboxylic acids that have been shown to be varied between human individuals [49]. Although no previous studies tested how ISV infections affect the host-seeking behaviour of mosquito vectors, the effect of arbovirus infections, especially DENV, has been relatively well examined [50–53]. However, the impact of DENV on the attraction to the host is still controversial, presumably due to the different experimental settings in each study. DENV infection has been shown to increase the host-seeking activity and the antennal response to synthetic human odorants in *Ae. aegypti* females 14–16 DPI; and chemosensory or neuromodulatory gene expressions in the antennae at 14 DPI [51]. A previous study showed CFAV replicated in the head of *Ae. aegypti* [23]. CFAV infection might induce a similar transcriptomic modulation related to the host-seeking response as DENV-1 infection. Nevertheless, an increase in the proportion of attracted mosquitoes in the CFAV-infected group was only observed in a particular experiment. Most of our assays showed CFAV infection had no significant impact on the human host-seeking ability. Even if CFAV would modulate gene expressions related to detecting odorant or thermal cues, the effect was not detectable in most cases in the assays. For instance, the distance from human arms could be too short to see the impact of CFAV. Further investigation is necessary to determine the impact of CFAV infections on human host-seeking ability in different experimental settings.

### 3.4. The number of blood-fed mosquitoes was not influenced by CFAV using artificial blood feeder

**Figure 4.**
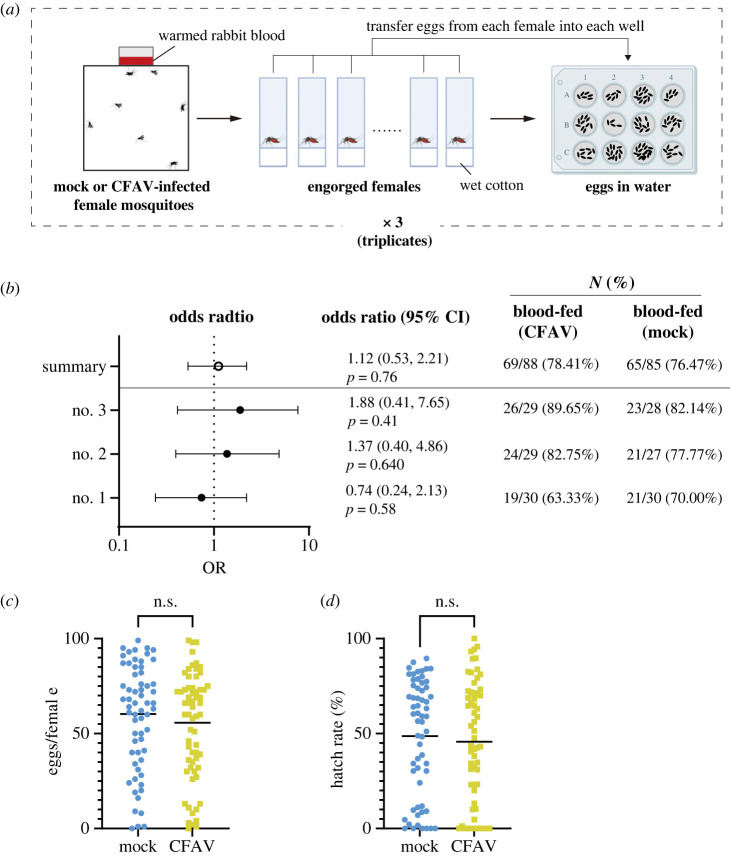
Blood intake, egg number and hatch rate were not dramatically impacted by CFAV infection in *Ae. aegypti*. (*a*) Schematic illustration of the experiment flow; blood-feeding was performed with artificial feeding system, each of the engorged females were isolated in a vial with wet cotton, the laid eggs were transferred to a 12-well plate. (*b*) Odds ratios for the relative impact of CFAV infection on the number of blood-fed mosquitoes. Forest plots represent the odds ratios of each replicate (no. 1 – no. 3) and the summarized value (Summary) with 95% confidence interval (CI). The number of CFAV- or mock-infected used and the proportion of blood-fed mosquitoes were shown in the right panel. (*c*) The number of eggs laid by an individual female and (*d*) their hatch rate. Bars represent mean. n.s., not significant (Mann-Whitney *U* test).

To examine if CFAV infection affects the ingestion of blood, we counted the number of blood-fed mosquitoes after providing rabbit blood through an artificial blood feeder (figure 4*a*, left). In three sets of experiments for CFAV- or mock-inoculated *Ae. aegypti* females, we did not observe a consistent difference in the number of blood-fed mosquitoes between the two groups (figure 4*b*). In summary, 78.4% (69 out of 88 individuals) and 76.47% (65 out of 85 individuals) were fed in CFAV-infected and mock-infected groups, respectively (figure 4*a*). Based on the pooled data from all experiments, the odds ratio (OR) was 1.12 (95% confidence interval, 0.53–2.21; *p*-value = 0.76), which suggested no significant association between CFAV infection and blood intake (figure 4*b*). Blood intake can be affected by the physical contact of the legs and taste in addition to host-seeking behaviour using thermal and odour cues. A recent study suggested that *Ae. aegypti* mosquitoes sense insect repellent with the tarsal segments of the legs [54]. The stylet neurons in *Ae. aegypti* females have been shown to detect the taste of blood [55]. The artificial blood-feeding apparatus in this study uses a 37^o^C-warmed metal feeder containing rabbit blood covered with pig intestine. CFAV infection seemed to have no effect on the sensory and taste systems of *Ae. aegypti* females in the laboratory setting using a non-human blood-feeding system.

### 3.5. CFAV-infected females showed similar reproductive abilities to uninfected

To assess the potential impacts of CFAV infection on mosquito reproduction, we measured the number of eggs per female and hatch rate using the engorged females from the artificial blood-feeding experiment (figure 4*a*). We observed a few mosquitoes were dead during the isolation phase in both groups. No significant difference was observed in egg number and hatch rate between CFAV-infected and mock-infected mosquitoes (figure 4*c,d*). To our knowledge, no study examined the effect of ISVs on mosquito reproduction. The presence of naturally infected laboratory mosquito colonies with AEFV, CFAV or CxFV implies that these ISV infections did not drastically reduce the fecundity or fertility in the laboratory conditions [11,20,21,29,30]. Our results were consistent with these observations. CFAV has been shown to infect *Ae. aegypti* ovaries, and is vertically transmitted by the intrathoracic injection [22,23]. CFAV replication kinetics showed that the highest range of viral RNA copies were detected at 15 DPI (figure 1*a*), when we counted the number of eggs. These suggested that no evident effect of CFAV on the reproductive ability in this study was unlikely due to the absence of infection in the ovaries. In the case of arboviruses, several studies assessed the impact of DENV, ZIKV or CHIKV on the fecundity and fertility in *Ae. aegypti* mosquitoes [35,37,38,41,56–58]. The effects are controversial especially for ZIKV and CHIKV, presumably because of the experimental design in each study such as the strain of mosquitoes and viruses used, the age of mosquitoes, and the number of oviposition [35,37,41,56,58]. Nevertheless, these arbovirus infections induce a fitness cost on mosquito reproduction. Unlike arboviruses, ISVs including CFAV are considered to be primarily maintained by transovarial vertical transmission [11,21,30,59]. CFAV may have become less pathogenic in the reproductive organs for their maintenance in the course of coevolution with *Ae. aegypti* mosquitoes. Further studies with various ISVs must be done to support this hypothesis conclusively.

### 3.6. Implications for potential use of CFAV in controlling arbovirus transmission

The use of symbiotic microbes to control arbovirus transmission from mosquito vectors to humans has gained great attention, well-exemplified by a symbiotic bacterium, *Wolbachia* [60]. Using the microbes as anti-arbovirus transmission strategies can be divided into two ways; (1) induce high fitness costs to reduce the mosquito population and the biting activity, (2) suppress the arboviral replication in the mosquito. To consider the potential use of ISVs for all these strategies, an understanding of the basic biological features of ISVs, including fitness costs, is necessary. The most influential parameters of the vectorial capacity of mosquitoes are daily survival and biting rate. If an ISV significantly enhances the biting frequency, this ISV may not be a great candidate as a biocontrol agent, for instance. This study assessed the potential impact of CFAV infection on *Ae. aegypti* mosquito fitness in the form of survival, blood-feeding behaviour, and reproduction. Overall, we observed that CFAV did not show drastic reduction or increase of the fitness-related traits tested. This viral tolerance of CFAV-infected mosquitoes is advantageous for spreading their infections into the mosquito population targeted for arbovirus blocking strategy. One exception is the observed enhancement of human host-seeking activity in a single experiment out of eight trials. Higher host-seeking activity can lead to more efficient arbovirus transmission from mosquitoes to humans. This observation must be further investigated to estimate the risk against the benefit of using CFAV. The human arm proximity assay used in this study focused on the mosquito attraction in a short range wherein mosquitoes can easily sense the host; however, further assessments with a longer distance from human hosts would be needed to provide more concrete evidence of the impact of CFAV in the host-seeking ability in the future.

CFAV showed similar replication kinetics between female and male mosquitoes. This finding can support the idea of spreading CFAV into natural *Ae. aegypti* population by releasing male adults that do not transmit arboviruses. The evidence of venereal transmission of CFAV from male to female adults using the naturally infected *Ae. aegypti* colony underpins that the release of infected males can efficiently expand CFAV prevalence [21]. This study focused on the intrathoracically injected *Ae. aegypti* to minimize the potential effects of genetic background on fitness. However, the route of infection would affect tissue tropism and/or replication levels of CFAV, both of which can influence mosquito fitness. Future studies would need to consider this aspect to examine the potential impact of ISVs on mosquito fitness.

## 4. Conclusion

ISVs in mosquito vectors have been of great interest in controlling arbovirus transmission. For the field application, it is a prerequisite to understand the potential risks of the spread of ISVs into the natural mosquito population such as increase in longevity and biting rate. In our study, we used CFAV and *Ae. aegypti* to understand the potential impacts of ISV infection on mosquito fitness including its survival, blood-feeding behaviour, and reproductive ability. Our results suggest both female and male *Ae. aegypti* are highly tolerant to CFAV infection, which is beneficial for the viruses to spread among the wild mosquito population as a biological agent, resulting in better control of arbovirus transmission between mosquitos and humans. Nevertheless, this study provides the first insight into how an ISV affects *Aedes* mosquito fitness and behaviour, leading to better understanding of mosquito-virus interactions and virus tolerance.

## Data Availability

Table S1, figure S1 and all data to generate figures are provided as electronic supplementary material. The data provided in electronic supplementary material is available in [61].
